# Unilateral psoas muscle sarcopenic indices, all-cause mortality, and novel cardiovascular events in patients undergoing hemodialysis

**DOI:** 10.1007/s40620-025-02450-y

**Published:** 2025-10-19

**Authors:** Takahiro Yajima, Maiko Arao

**Affiliations:** Department of Nephrology, Matsunami General Hospital, Gifu, 501-6062 Japan

**Keywords:** Computed tomography, Psoas muscle sarcopenic indices, Mortality, Cardiovascular event, Hemodialysis

Computed tomography (CT)-measured sarcopenia indices have emerged as surrogate markers of sarcopenia and mortality indicators in patients on hemodialysis [1–5]. Recently, we demonstrated that bilateral psoas muscle sarcopenia indices, such as the psoas muscle index and psoas muscle gauge measured at the 4th vertebral level, were independently associated with an increased risk of cardiovascular events and all-cause mortality in these patients [6]. We previously reported that unilateral (right) psoas muscle thickness adjusted for height (a surrogate marker of muscle quantity reflecting bioimpedance that can estimate whole-body skeletal muscle mass volume) was a significant predictor of all-cause and cardiovascular mortality in patients undergoing hemodialysis [7]. We hypothesized that assessing the unilateral psoas muscle could be useful in predicting these clinical outcomes, although some previous studies have shown that psoas muscles are anatomically asymmetrical due to muscle degeneration caused by lumbar spinal stenosis and hip osteoarthritis [8, 9]. Thus, we compared the differences in the psoas muscle sarcopenia indices between the right and left sides of the psoas muscle. Additionally, we also compared the predictability of bilateral and unilateral psoas muscle sarcopenic indices for all-cause mortality and novel cardiovascular events in patients undergoing hemodialysis.

The present study was conducted as a secondary analysis of previous research examining the associations of psoas muscle index and psoas muscle gauge with adverse clinical outcomes [6]. The details of the methods we used are described in the Supplementary Methods.

After excluding 25 patients with a known history of cancer, the study included 217 patients undergoing maintenance hemodialysis (male: 66.4%; mean age: 62.7 years; median hemodialysis vintage: 22 months; history of cardiovascular disease: 63.7%; history of symptomatic lumbar spinal stenosis: *N* = 12; hip osteoarthritis: *N* = 2) (Supplementary Table 1).

Two blinded investigators (MA and TY) measured the psoas muscle area and psoas muscle density of 30 randomly selected patients to investigate reproducibility. The intra- and inter-observer intraclass correlation coefficients (ICCs) were > 0.90 (Supplementary Table 2). In addition, Bland–Altman analysis revealed the consistency of the two measurements (Supplementary Figs. 2–4).

Excellent agreements of psoas muscle area and psoas muscle density were found between right and left psoas muscles (Supplementary Table 3, Supplementary Fig. 5). Additionally, psoas muscle area and psoas muscle density were comparable between the right and left psoas muscles (Supplementary Table 3). Significant correlations were identified between the right and left psoas muscle sarcopenic indices (Supplementary Figs. 6, 7), as well as between the right or left and bilateral psoas muscle sarcopenic indices (Supplementary Figs. 8–11).

During a median follow-up period of 4.4 (2.4–7.3) years, 95 patients experienced novel cardiovascular events, and 85 died (cardiovascular death: 55.3%). The C-indexes for predicting all-cause mortality significantly improved after adding psoas muscle index (right, 0.808; left, 0.816; bilateral, 0.816) and psoas muscle gauge (right, 0.857; left, 0.855; bilateral, 0.855) to the baseline risk model (0.746) (Table 1). Moreover, the C-indexes of either right, left, or bilateral psoas muscle gauge were higher than those of either right, left, or bilateral psoas muscle index (Table 1). Similar results were obtained for novel cardiovascular events (Table 1). These trends were also observed in patients without a history of lumbar spinal stenosis or hip osteoarthritis (Table 1).

**Table 1:** C-index comparisons among psoas muscle sarcopenic indices for predicting all-cause mortality and novel cardiovascular events

Variable	*C*-index	*P*-value					
All patients (*N* = 217)							
*All-cause mortality*							
Baseline risk model	0.746 (0.680–0.813)	Ref					
+ Right psoas muscle index	0.808 (0.748–0.868)	0.0073	Ref				
+ Left psoas muscle index	0.816 (0.759–0.873)	0.0033	0.55	Ref			
+ Bilateral psoas muscle index	0.816 (0.759–0.873)	0.0033	0.55	1.0	Ref		
+ Right psoas muscle gauge	0.857 (0.806–0.908)	0.00018	0.0023	0.037	0.037	Ref	
+ Left psoas muscle gauge	0.855 (0.806–0.905)	0.00010	0.0095	0.0017	0.0017	0.91	Ref
+ Bilateral psoas muscle gauge	0.855 (0.806–0.905)	0.00010	0.0095	0.0017	0.0017	0.91	1.0
*Novel cardiovascular events*							
Baseline risk model	0.743 (0.678–0.809)	Ref					
+ Right psoas muscle index	0.780 (0.718–0.843)	0.043	Ref				
+ Left psoas muscle index	0.789 (0.728–0.851)	0.025	0.44	Ref			
+ Bilateral psoas muscle index	0.789 (0.728–0.851)	0.025	0.44	1.0	Ref		
+ Right psoas muscle gauge	0.810 (0.751–0.869)	0.0042	0.0083	0.18	0.18	Ref	
+ Left psoas muscle gauge	0.806 (0.748–0.865)	0.0059	0.060	0.073	0.073	0.77	Ref
+ Bilateral psoas muscle gauge	0.806 (0.748–0.865)	0.0059	0.060	0.073	0.073	0.77	1.0
Patients without lumbar spinal stenosis or hip osteoarthritis (*N* = 203)							
*All-cause mortality*							
Baseline risk model	0.742 (0.673–0.811)	Ref					
+ Right psoas muscle index	0.802 (0.739–0.865)	0.012	Ref				
+ Left psoas muscle index	0.805 (0.745–0.865)	0.0099	0.83	Ref			
+ Bilateral psoas muscle index	0.812 (0.752–0.872)	0.0048	0.81	0.41	Ref		
+ Right psoas muscle gauge	0.847 (0.793–0.902)	0.00060	0.0061	0.045	0.038	Ref	
+ Left psoas muscle gauge	0.845 (0.793–0.898)	0.00041	0.028	0.0030	0.023	0.89	Ref
+ Bilateral psoas muscle gauge	0.851 (0.798–0.903)	0.00038	0.0051	0.0072	0.0093	0.67	0.54
*Novel cardiovascular events*							
Baseline risk model	0.770 (0.705–0.835)	Ref					
+ Right psoas muscle index	0.809 (0.749–0.870)	0.033	Ref				
+ Left psoas muscle index	0.823 (0.765–0.881)	0.011	0.25	Ref			
+ Bilateral psoas muscle index	0.818 (0.760–0.877)	0.015	0.13	0.50	Ref		
+ Right psoas muscle gauge	0.837 (0.800–0.893)	0.0054	0.022	0.40	0.16	Ref	
+ Left psoas muscle gauge	0.837 (0.782–0.893)	0.0043	0.054	0.14	0.098	0.93	Ref
+ Bilateral psoas muscle gauge	0.839 (0.783–0.895)	0.0040	0.019	0.20	0.066	0.70	0.82

*Ref* reference

In this study, the reproducibility of the psoas muscle area and psoas muscle density measurements was excellent. In addition, the agreement between the right and left psoas muscles of the psoas muscle area and psoas muscle density were excellent. Very strong correlations were also found between either right or left and bilateral psoas muscle sarcopenic indices in patients undergoing hemodialysis. Moreover, the C-indexes for predicting all-cause mortality and novel cardiovascular events similarly and significantly improved after adding either right, left, or bilateral psoas muscle sarcopenic indices into the baseline risk model in these patients; the performance of psoas muscle gauge, an integrated sarcopenic indicator derived from psoas muscle index (a muscle quantity indicator) and psoas muscle density (a muscle quality indicator), was superior to that of psoas muscle index [1, 2, 6]. Psoas muscle gauge may reflect muscle quality in addition to muscle quantity, therefore, psoas muscle gauge may be superior to psoas muscle index in predicting mortality and cardiovascular events, as previously reported [6]. These findings suggest that assessing unilateral psoas muscle sarcopenic indices may be a simplified and potentially accessible approach with predictive values for mortality and cardiovascular events similar to those of bilateral psoas muscle sarcopenic indices. However, we believe that further validation is required to evaluate its usefulness in clinical practice, with additional evidence regarding its feasibility, cost, time reduction, and implementation barriers.

This study had several limitations. First, this single-center retrospective study enrolled only Japanese patients undergoing hemodialysis. Second, a confirmed reference range for unilateral psoas muscle sarcopenic indices has not been reported; only a recommended cutoff range for bilateral psoas muscle sarcopenic indices of the mean ± 2 standard deviations in Asian populations has been established. Third, atrophy of the psoas muscle, a spinal stabilization muscle, has been reported as a clinical manifestation of lumbar spinal stenosis resulting from neurogenic compromise of the lumbar 2–4 spinal roots and hip osteoarthritis due to restricted hip joint movement [8, 9]. Psoas muscle atrophy, which manifests as asymmetrical muscle volume reduction and morphological changes with fatty degeneration, may influence the CT-measured psoas muscle area and CT attenuation. In the present study, only 14 patients had symptomatic lumbar spinal stenosis or hip osteoarthritis. Judging from the visualized outlier from the Bland–Altman analysis in patients without these diseases, some other causes of unrecognized psoas muscle asymmetry may exist; this consideration might lead to selecting the better side of the psoas muscle in each patient (i.e., dominant vs. non-dominant or affected vs. unaffected). Therefore, further prospective, multicenter, and large-scale studies that include other ethnic groups with ethnicity-specific reference values for the young population and possible selection of the optimal side of the psoas muscle are required to establish and generalize the clinical utility of unilateral psoas muscle sarcopenic indices.

## Supplementary Information

Below is the link to the electronic supplementary material.

Supplementary file 1 (DOCX 19 kb)

Supplementary file 2 (DOCX 22 kb)

Supplementary file 3 (DOCX 16 kb)

Supplementary file 4 (DOCX 16 kb)

Supplementary file 5 Supplementary Figure 1. Example of measuring the psoas muscle area and average computed tomography value using ImageJ in a 78-year-old man. We selected a single cross-sectional slice of a computed tomography (CT) image at the lumbar vertebral 4th level (a). We used ImageJ (National Institutes of Health) with the “Polygon selection tool” to trace the outer perimeter of the psoas muscles (b, c). We set the lower and upper thresholds of the CT values to −29 and + 150 Hounsfield unit (HU), respectively. We defined the selected area (red) with a yellow line as the right psoas area (cm^2^) and the average CT value of the area as the average right psoas muscle density (HU), respectively (d). Similarly, we obtained the left psoas muscle area and psoas muscle density (e). In the present case, the right psoas muscle area and density were 5.7 cm^2^ and 38.1 HU, respectively, and the left was 9.2 cm^2^ and 42.7 HU, respectively. We defined the psoas muscle index as psoas muscle area/height^2^ (cm^2^/m^2^). We defined bilateral psoas muscle density as the mean CT value of the right and left psoas muscles [ = (average right psoas muscle density × right psoas muscle area + average left psoas muscle density × left psoas muscle area) / (right psoas muscle area + left psoas muscle area)] (HU). (TIFF 1052 kb)

Supplementary file 6 Supplementary Figure 2. Bland-Altman plots showing agreement of the psoas muscle area and density at trial 1 and trial 2 by MA. PMA, psoas muscle area; PMD, psoas muscle density. (TIFF 254 kb)

Supplementary file 7 Supplementary Figure 3. Bland-Altman plots showing agreement of the psoas muscle area and density at trial 1 and trial 2 by TY. PMA, psoas muscle area; PMD, psoas muscle density. (TIFF 258 kb)

Supplementary file 8 Supplementary Figure 4. Bland-Altman plots showing agreement of the psoas muscle area and density MA and TY. PMA, psoas muscle area; PMD, psoas muscle density. (TIFF 256 kb)

Supplementary file 9 Supplementary Figure 5. Bland-Altman plots showing agreement of the psoas muscle area and density in all patients (a,b), in those without lumbar spinal stenosis or hip osteoarthritis (c,d), and in those with lumbar spinal stenosis or hip osteoarthritis (e,f). PMA, psoas muscle area; PMD, psoas muscle density. (TIFF 357 kb)

Supplementary file 10 Supplementary Figure 6. Correlations between the right and left psoas muscle sarcopenic indices in all patients. AU, arbitrary unit; HU, Hounsfield units; PMA, psoas muscle area; PMD, psoas muscle density; PMG, psoas muscle gauge; PMI, psoas muscle index. (TIFF 150 kb)

Supplementary file 11 Supplementary Figure 7. Correlations between the right and left psoas muscle sarcopenic indices in patients with (red) or without (blue) lumbar spinal stenosis or hip osteoarthritis. AU, arbitrary unit; HU, Hounsfield units; PMA, psoas muscle area; PMD, psoas muscle density; PMG, psoas muscle gauge; PMI, psoas muscle index. (TIFF 445 kb)

Supplementary file 12 Supplementary Figure 8. Correlations between the right and bilateral psoas muscle sarcopenic indices in all patients. AU, arbitrary unit; HU, Hounsfield units; PMA, psoas muscle area; PMD, psoas muscle density; PMG, psoas muscle gauge; PMI, psoas muscle index. (TIFF 132 kb)

Supplementary file 13 Supplementary Figure 9. Correlations between the right and bilateral psoas muscle sarcopenic indices in patients with (red) or without (blue) lumbar spinal stenosis or hip osteoarthritis. AU, arbitrary unit; HU, Hounsfield units; PMA, psoas muscle area; PMD, psoas muscle density; PMG, psoas muscle gauge; PMI, psoas muscle index. (TIFF 400 kb)

Supplementary file 14 Supplementary Figure 10. Correlations between the left and bilateral psoas muscle sarcopenic indices in all patients. AU, arbitrary unit; HU, Hounsfield units; PMA, psoas muscle area; PMD, psoas muscle density; PMG, psoas muscle gauge; PMI, psoas muscle index. (TIFF 132 kb)

Supplementary file 15 Supplementary Figure 11. Correlations between the left and bilateral psoas muscle sarcopenic indices in patients with (red) or without (blue) lumbar spinal stenosis or hip osteoarthritis. AU, arbitrary unit; HU, Hounsfield units; PMA, psoas muscle area; PMD, psoas muscle density; PMG, psoas muscle gauge; PMI, psoas muscle index. (TIFF 394 kb)

## Data Availability

The datasets generated and/or analyzed in the present study are available from the corresponding author upon reasonable request.
