# Supplementary material for: Unilateral psoas muscle sarcopenic indices, all-cause mortality, and novel cardiovascular events in patients undergoing hemodialysis
Source: J Nephrol. 2025 Oct 19;38(9):3045–7. doi: 10.1007/s40620-025-02450-y (PMC12712098; doi:10.1007/s40620-025-02450-y)
Supplement: Supplementary file 1 — (DOCX 19 kb) [file 40620_2025_2450_MOESM1_ESM.docx]

**Supplementary Methods**

Patients undergoing maintenance hemodialysis were enrolled between January 2008 and December 2019 and followed up until December 2021 at the outpatient clinic. ImageJ version 1.53 (National Institutes of Health, Bethesda, MD, USA, https://imagej.nih.gov/ij/) was used to measure the area (cm^2^) and mean CT value (in Hounsfield units [HU]) of the bilateral psoas muscles at the L4 vertebral body level, as previously reported [1,2] (Supplementary Figure 1). The right, left, and bilateral psoas muscle areas and mean CT values were defined as right, left, and bilateral PMAs and PMDs, respectively. PMI (cm^2^/m^2^) was defined as PMA (cm^2^)/height^2^. PMG (arbitrary units [AUs]) was defined as PMI × PMD (HU). To investigate the reproducibility of intra- and inter-rater measurements of PMA and PMD, the intraclass correlation coefficient (ICC) and Bland-Altman analyses were used. An ICC larger than 0.90 was considered clinically acceptable and indicative of excellent reproducibility. To assess the agreement of psoas muscle sarcopenic indices between the right and left psoas muscles, ICCs and Bland-Altman analyses were performed. In addition, correlations and differences among the right, left, and bilateral psoas muscle sarcopenic indices were examined using Pearson’s correlation coefficient and paired t-test or Wilcoxon signed-rank test, respectively. Moreover, the multivariable-adjusted C-indexes, defined as the areas under the receiver operating characteristic curves, were compared using the DeLong test for predicting all-cause mortality and novel cardiovascular events. The baseline risk model included age, sex, history of cardiovascular disease, C-reactive protein level, simplified creatinine index, and geriatric nutritional risk index, as described in the primary study [6]. These analyses were performed in all patients and in those with or without a history of lumbar spinal stenosis or hip osteoarthritis. In the present study, only the available data were used for analysis; therefore, no imputation of missing data was performed. For statistical analysis, SPSS Statistics Version 31 (IBM Corp., Armonk, N.Y., USA) and R Version 4.3.2. Statistical significance was set at p < 0.05.

1. Arao M, Yajima T (2024) Computed tomography-based abdominal sarcopenic indices and bio-impedance analysis-based skeletal muscle mass index in hemodialyzed patients. Clin Nutr ESPEN 59:21-28. https://doi.org/10.1016/j.clnesp.2023.11.012.
2. Yajima T, Arao M (2025) Computed tomography-based abdominal myosteatosis indicators and handgrip strength in hemodialyzed patients. J Ren Nutr [In Press] https://doi.org/10.1053/j.jrn.2025.04.001.
3. Yajima T, Arao M (2025) Psoas muscle gauge and adverse clinical outcomes in patients on hemodialysis. J Nephrol 38:655-664. https://doi.org/10.1007/s40620-024-02191-4.
