# Supplementary material for: Unilateral psoas muscle sarcopenic indices, all-cause mortality, and novel cardiovascular events in patients undergoing hemodialysis
Source: J Nephrol. 2025 Oct 19;38(9):3045–7. doi: 10.1007/s40620-025-02450-y (PMC12712098; doi:10.1007/s40620-025-02450-y)
Supplement: Supplementary file 2 — (DOCX 22 kb) [file 40620_2025_2450_MOESM2_ESM.docx]

Supplementary Table 1. Background characteristics of the study participants

| Characteristic | All patients (*N* = 217) |
| --- | --- |
| Male (%) | 66.4 |
| Age (years) | 62.7 ± 13.7 |
| Underlying kidney disease |  |
| Diabetic kidney disease (%) | 42.9 |
| Chronic glomerulonephritis (%) | 30.0 |
| Nephrosclerosis (%) | 19.3 |
| Others (%) | 7.8 |
| Hemodialysis vintage (months) | 22.0 (10.8–60.0) |
| Diabetes (%) | 46.1 |
| Hypertension (%) | 94.5 |
| Cardiovascular disease history (%) | 65.0 |
| History of symptomatic lumbar spinal stenosis (%, *N*) | 5.5 (*N* = 12) |
| History of symptomatic hip osteoarthritis (%, *N*) | 0.9 (*N* = 2) |
| Hemoglobin (g/dL) | 10.5 ± 1.4 |
| Total cholesterol (mg/dL) | 150 ± 34 |
| Creatinine (mg/dL) | 9.4 ± 3.1 |
| C-reactive protein (mg/dL) | 0.16 (0.07–0.54) |
| Albumin (g/dL) | 3.7 ± 0.5 |
| Body mass index (kg/m^2^) | 22.2 ± 4.1 |
| Geriatric nutritional risk index | 93.5 ± 8.4 |
| Simplified creatinine index (mg/kg/day) | 20.6 ± 3.1 |
| Bilateral psoas muscle area (cm^2^) | 14.2 ± 6.1 |
| Right psoas muscle area (cm^2^) | 7.1 ± 3.1 |
| Left psoas muscle area (cm^2^) | 7.1 ± 3.1 |
| Bilateral psoas muscle index (cm^2^/m^2^) | 5.4 ± 2.0 |
| Right psoas muscle index (cm^2^/m^2^) | 2.7 ± 1.0 |
| Left psoas muscle index (cm^2^/m^2^) | 2.7 ± 1.0 |
| Bilateral psoas muscle density (HU) | 41.5 ± 9.4 |
| Right psoas muscle density (HU) | 41.2 ± 9.9 |
| Left psoas muscle density (HU) | 41.8 ± 9.7 |
| Bilateral psoas muscle gauge (AU) | 219.0 (149.3–311.6) |
| Right psoas muscle gauge (AU) | 109.7 (68.3–151.4) |
| Left psoas muscle gauge (AU) | 104.0 (73.5–161.6) |

AU, arbitrary unit; HU, Hounsfield unit.
