# Supplementary material for: Unilateral psoas muscle sarcopenic indices, all-cause mortality, and novel cardiovascular events in patients undergoing hemodialysis
Source: J Nephrol. 2025 Oct 19;38(9):3045–7. doi: 10.1007/s40620-025-02450-y (PMC12712098; doi:10.1007/s40620-025-02450-y)
Supplement: Supplementary file 3 — (DOCX 16 kb) [file 40620_2025_2450_MOESM3_ESM.docx]

Supplemental Table 2. Reproducibility of measuring the psoas muscle area and density.

| Variables | MA | | TY | | Intra-rater MA | Intra-rater TY | Inter-rater MA-TY |
| --- | --- | --- | --- | --- | --- | --- | --- |
|  | Trial 1 | Trial 2 | Trial 1 | Trial 2 | ICC (95%CI) | ICC (95%CI) | ICC (95%CI) |
| Right psoas muscle area (cm^2^) | 7.5 ± 2.6 | 7.5 ± 2.6 | 7.4 ± 2.5 | 7.4 ± 2.6 | 0.992 (0.983-0.996) | 0.998 (0.995-0.999) | 0.989 (0.977-0.995) |
| Right psoas muscle density (HU) | 32.9 ± 8.1 | 32.7 ± 8.1 | 33.1 ± 8.2 | 33.0 ± 8.1 | 0.992 (0.984-0.996) | 0.993 (0.994-0.996) | 0.983 (0.964-0.992) |
| Left psoas muscle area (cm^2^) | 7.7 ± 2.8 | 7.7 ± 2.8 | 7.6 ± 2.8 | 7.7 ± 2.8 | 0.997 (0.994-0.999) | 0.998 (0.985-0.999) | 0.995 (0.990-0.998) |
| Left psoas muscle density (HU) | 33.3 ± 8.5 | 33.2 ± 8.5 | 33.5 ± 8.6 | 33.4 ± 8.4 | 0.994 (0.987-0.997) | 0.992 (0.983-0.996) | 0.985 (0.969-0.993) |

ICC, intraclass correlation coefficient; 95%CI, 95% confidence interval; HU, Hounsfield Unit.
