# Supplementary material for: Unilateral psoas muscle sarcopenic indices, all-cause mortality, and novel cardiovascular events in patients undergoing hemodialysis
Source: J Nephrol. 2025 Oct 19;38(9):3045–7. doi: 10.1007/s40620-025-02450-y (PMC12712098; doi:10.1007/s40620-025-02450-y)
Supplement: Supplementary file 4 — (DOCX 16 kb) [file 40620_2025_2450_MOESM4_ESM.docx]

Supplementary Table 3. Agreements between right and left psoas muscle sarcopenic indices.

| Variables | Right psoas muscle | Left psoas muscle | *P*-value | ICC (95%CI) |
| --- | --- | --- | --- | --- |
| All patients (*N* = 217) |  |  |  |  |
| Psoas muscle area (cm^2^) | 7.1 ± 3.1 | 7.1 ± 3.1 | 0.81 | 0.964 (0.953-0.972) |
| Psoas muscle density (HU) | 41.2 ± 9.9 | 41.7 ± 9.7 | 0.084 | 0.929 (0.908-0.946) |
| Psoas muscle index (cm^2^/m^2^) | 2.7 ± 1.0 | 2.7 ± 1.0 | 0.84 | 0.950 (0.934-0.962) |
| Psoas muscle gauge (AU) | 109.7 (68.3–151.4) | 104.0 (73.5–161.6) | 0.19 | 0.958 (0.945-0.967) |
| Patients without symptomatic lumbar spinal stenosis or hip osteoarthritis (*N* = 203) |  |  |  |  |
| Psoas muscle area (cm^2^) | 7.2 ± 3.1 | 7.2 ± 3.1 | 0.73 | 0.963 (0.952-0.972) |
| Psoas muscle density (HU) | 41.5 ± 9.4 | 42.1 ± 9.0 | 0.054 | 0.920 (0.895-0.940) |
| Psoas muscle index (cm^2^/m^2^) | 2.7 ± 1.0 | 2.7 ± 1.0 | 0.76 | 0.949 (0.932-0.961) |
| Psoas muscle gauge (AU) | 113.1 (70.1–153.7) | 105.1 (75.4–162.3) | 0.24 | 0.960 (0.947-0.970) |
| Patients with symptomatic lumbar spinal stenosis or hip osteoarthritis (*N* = 14) |  |  |  |  |
| Psoas muscle area (cm^2^) | 6.3 ± 3.3 | 6.2 ± 3.0 | 0.73 | 0.965 (0.890-0.989) |
| Psoas muscle density (HU) | 36.2 ± 15.2 | 35.5 ± 15.4 | 0.64 | 0.968 (0.902-0.990) |
| Psoas muscle index (cm^2^/m^2^) | 2.5 ± 1.2 | 2.5 ± 1.2 | 0.72 | 0.962 (0.881-0.988) |
| Psoas muscle gauge (AU) | 78.0 (48.5–125.8) | 85.8 (39.5–125.8) | 0.62 | 0.939 (0.809-0.980) |

ICC, intraclass correlation coefficient; 95%CI, 95% confidence interval; HU, Hounsfield units; AU, arbitrary unit.
