# Supplementary figures and images for: Unilateral psoas muscle sarcopenic indices, all-cause mortality, and novel cardiovascular events in patients undergoing hemodialysis
Source: J Nephrol. 2025 Oct 19;38(9):3045–7. doi: 10.1007/s40620-025-02450-y (PMC12712098; doi:10.1007/s40620-025-02450-y)

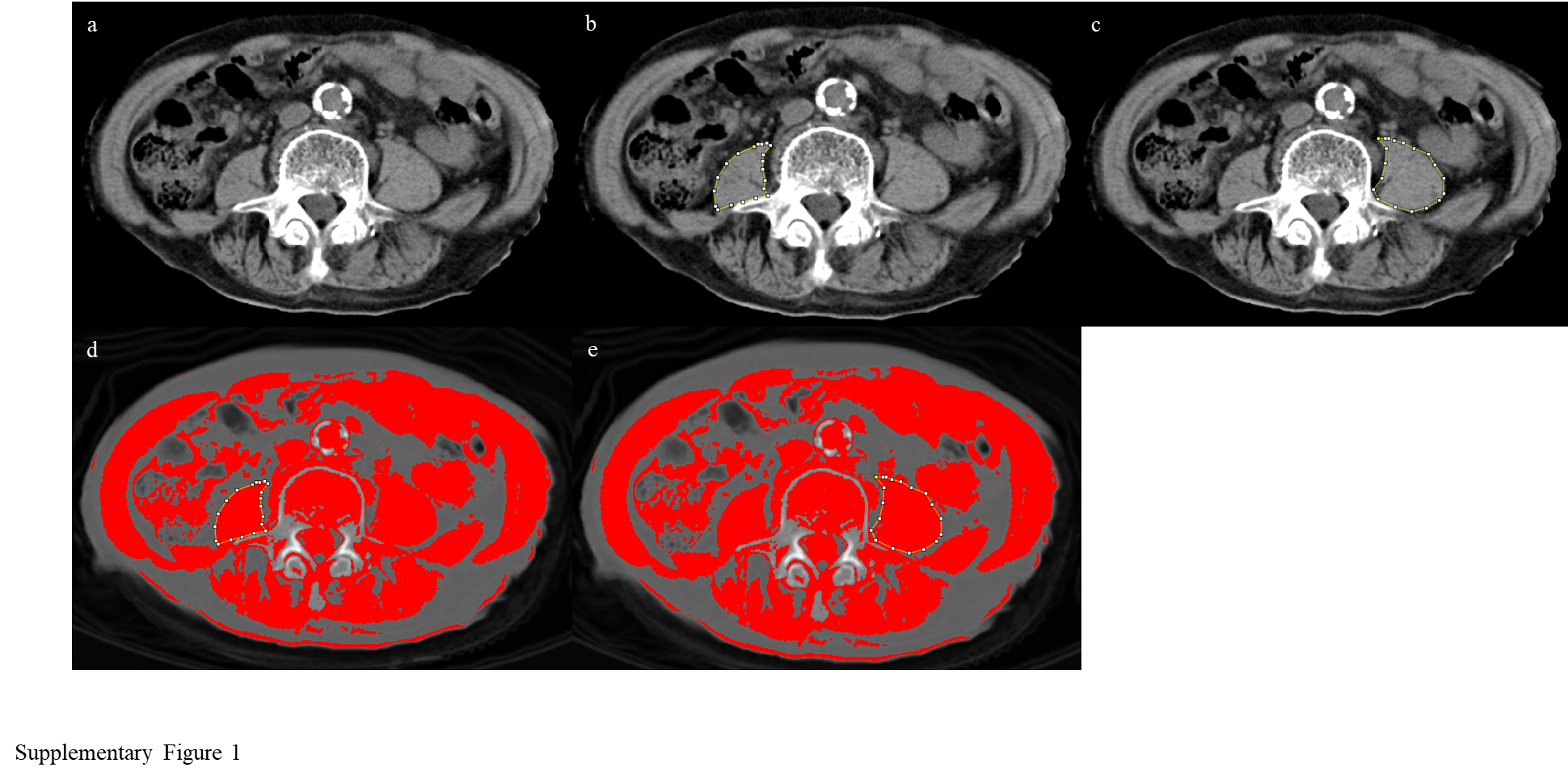

Supplement: Supplementary file 5 — Supplementary Figure 1. Example of measuring the psoas muscle area and average computed tomography value using ImageJ in a 78-year-old man. We selected a single cross-sectional slice of a computed tomography (CT) image at the lumbar vertebral 4th level (a). We used ImageJ (National Institutes of Health) with the “Polygon selection tool” to trace the outer perimeter of the psoas muscles (b, c). We set the lower and upper thresholds of the CT values to −29 and + 150 Hounsfield unit (HU), respectively. We defined the selected area (red) with a yellow line as the right psoas area (cm2) and the average CT value of the area as the average right psoas muscle density (HU), respectively (d). Similarly, we obtained the left psoas muscle area and psoas muscle density (e). In the present case, the right psoas muscle area and density were 5.7 cm2 and 38.1 HU, respectively, and the left was 9.2 cm2 and 42.7 HU, respectively. We defined the psoas muscle index as psoas muscle area/height2 (cm2/m2). We defined bilateral psoas muscle density as the mean CT value of the right and left psoas muscles [ = (average right psoas muscle density × right psoas muscle area + average left psoas muscle density × left psoas muscle area) / (right psoas muscle area + left psoas muscle area)] (HU). (TIFF 1052 kb) [file 40620_2025_2450_MOESM5_ESM.tiff]

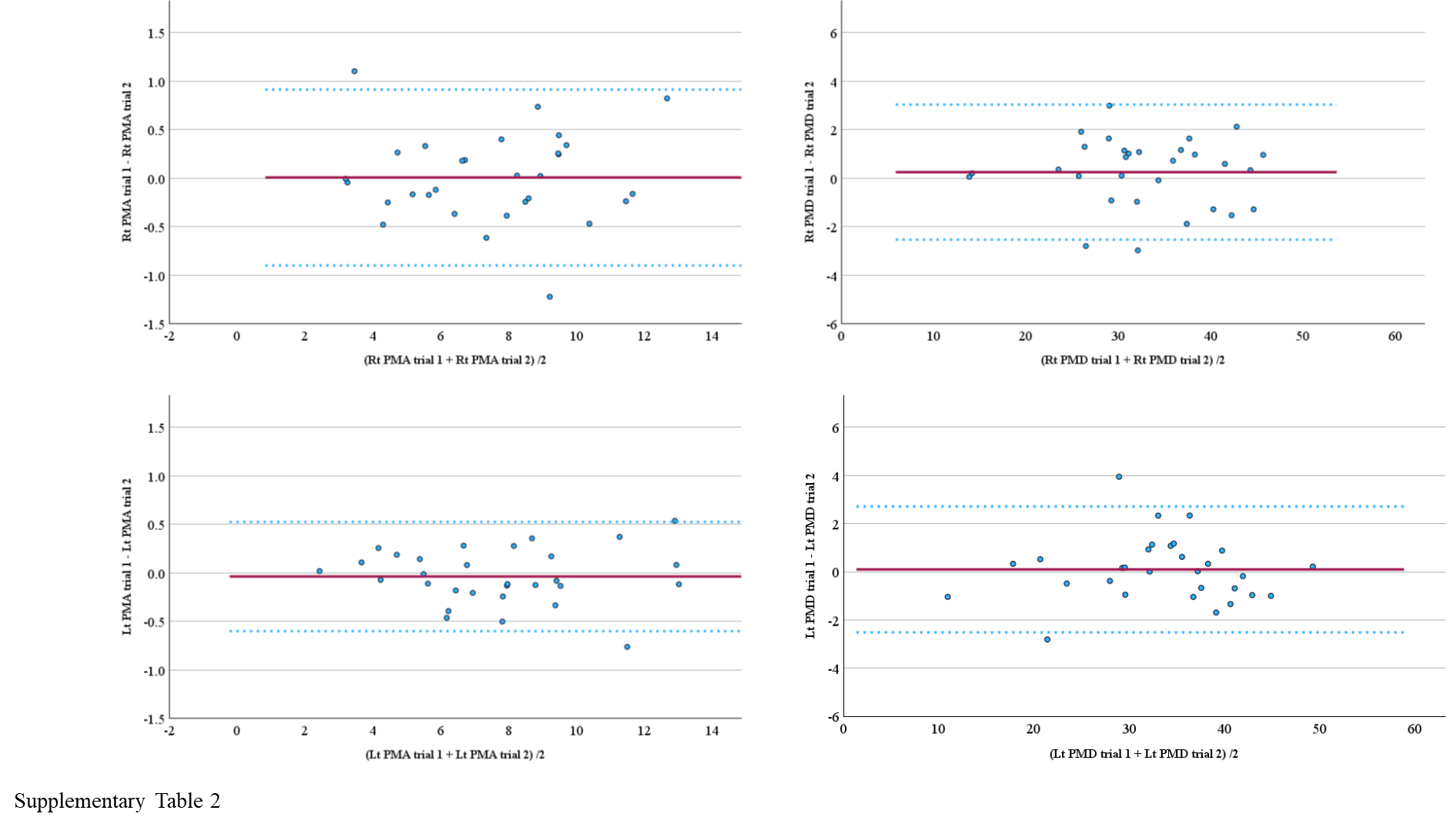

Supplement: Supplementary file 6 — Supplementary Figure 2. Bland-Altman plots showing agreement of the psoas muscle area and density at trial 1 and trial 2 by MA. PMA, psoas muscle area; PMD, psoas muscle density. (TIFF 254 kb) [file 40620_2025_2450_MOESM6_ESM.tiff]

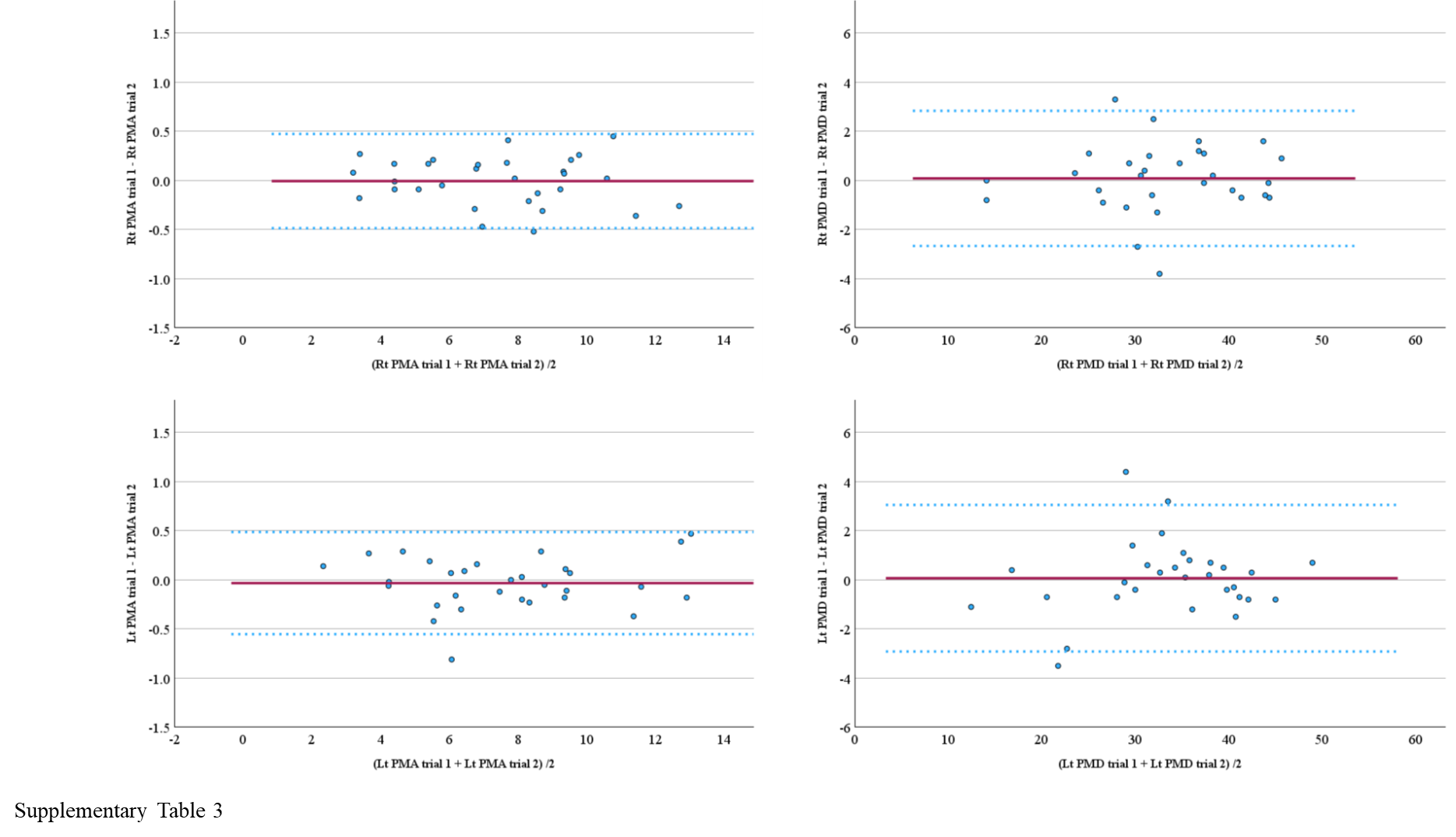

Supplement: Supplementary file 7 — Supplementary Figure 3. Bland-Altman plots showing agreement of the psoas muscle area and density at trial 1 and trial 2 by TY. PMA, psoas muscle area; PMD, psoas muscle density. (TIFF 258 kb) [file 40620_2025_2450_MOESM7_ESM.tiff]

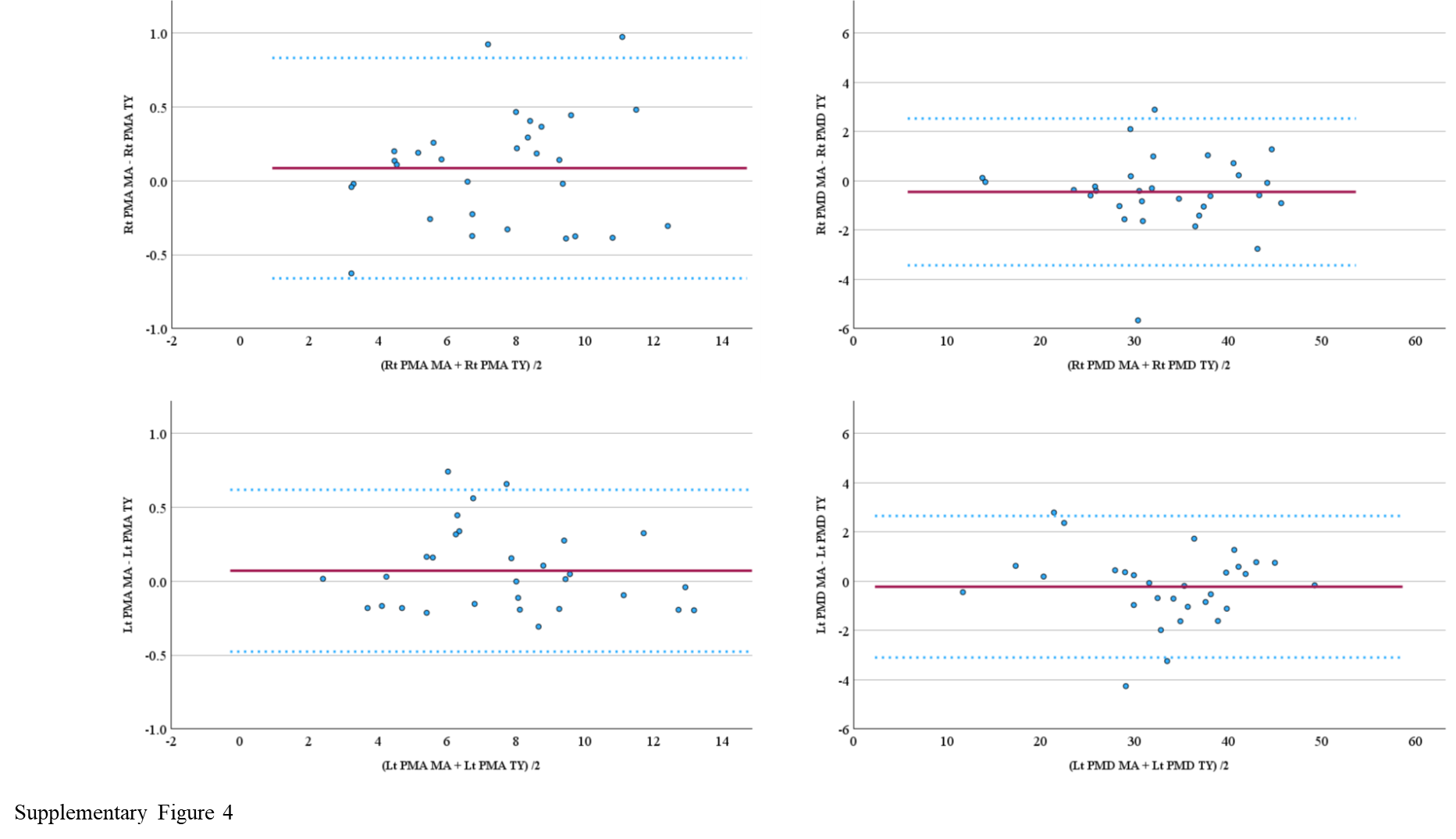

Supplement: Supplementary file 8 — Supplementary Figure 4. Bland-Altman plots showing agreement of the psoas muscle area and density MA and TY. PMA, psoas muscle area; PMD, psoas muscle density. (TIFF 256 kb) [file 40620_2025_2450_MOESM8_ESM.tiff]

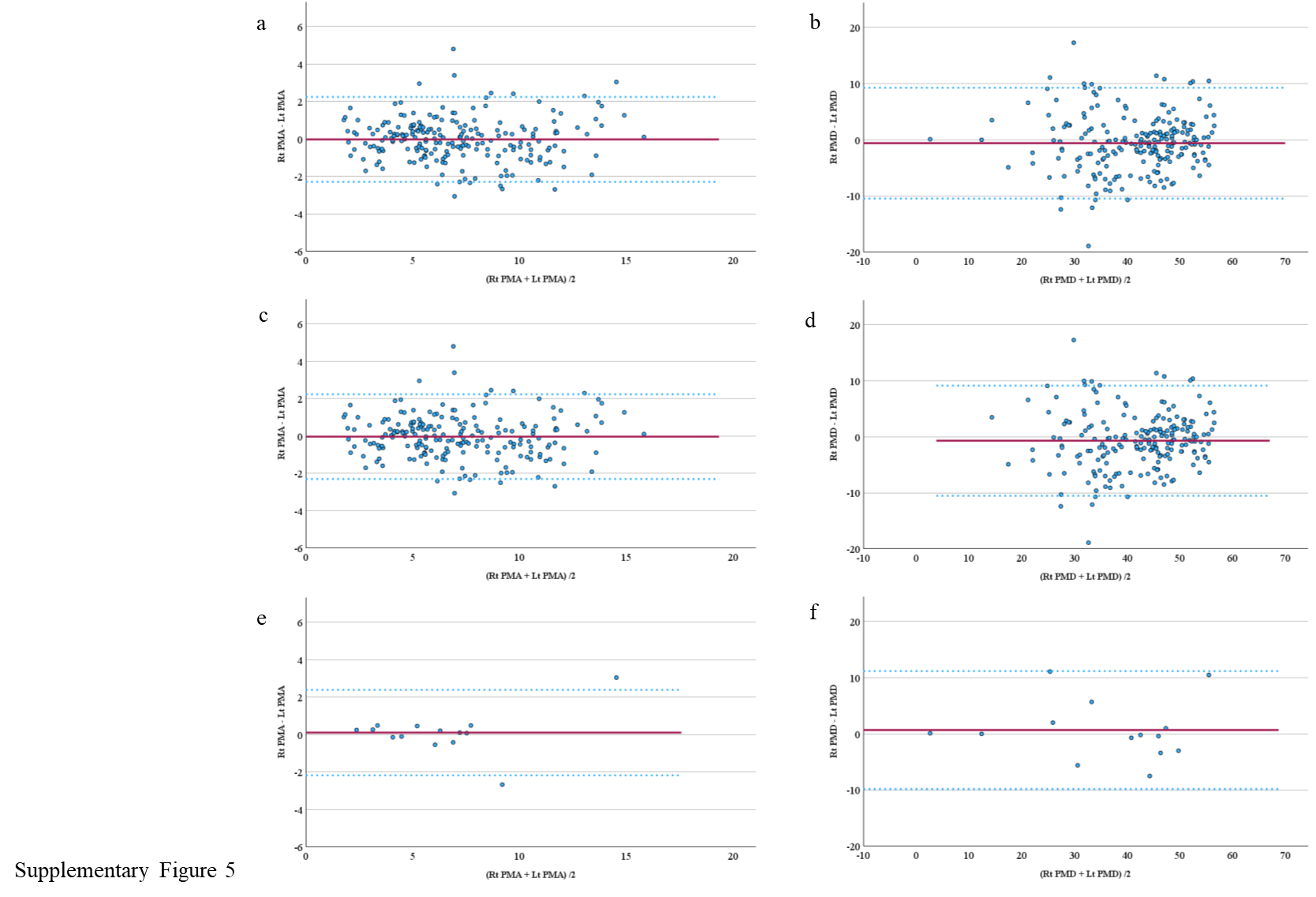

Supplement: Supplementary file 9 — Supplementary Figure 5. Bland-Altman plots showing agreement of the psoas muscle area and density in all patients (a,b), in those without lumbar spinal stenosis or hip osteoarthritis (c,d), and in those with lumbar spinal stenosis or hip osteoarthritis (e,f). PMA, psoas muscle area; PMD, psoas muscle density. (TIFF 357 kb) [file 40620_2025_2450_MOESM9_ESM.tiff]

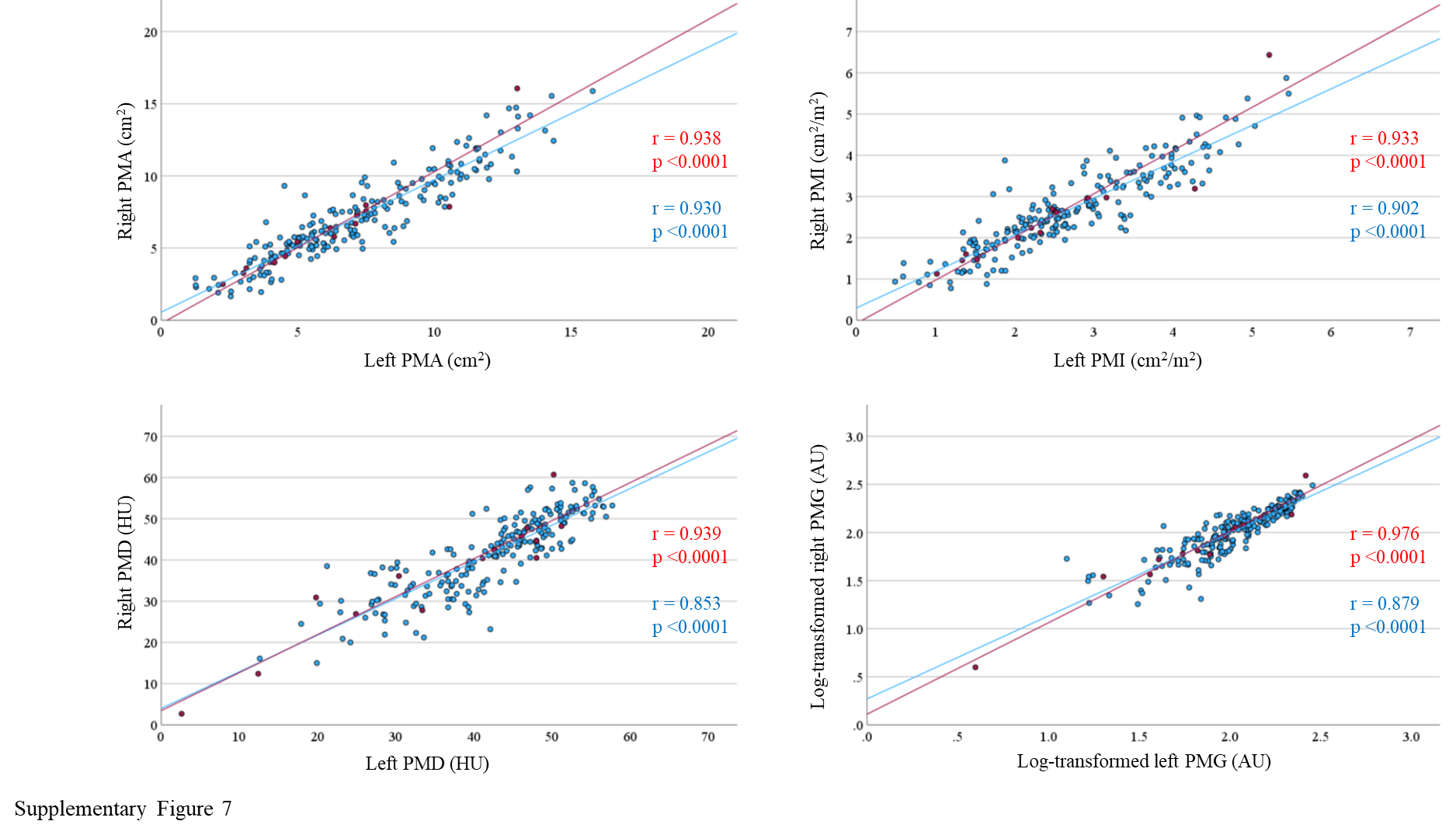

Supplement: Supplementary file 11 — Supplementary Figure 7. Correlations between the right and left psoas muscle sarcopenic indices in patients with (red) or without (blue) lumbar spinal stenosis or hip osteoarthritis. AU, arbitrary unit; HU, Hounsfield units; PMA, psoas muscle area; PMD, psoas muscle density; PMG, psoas muscle gauge; PMI, psoas muscle index. (TIFF 445 kb) [file 40620_2025_2450_MOESM11_ESM.tiff]

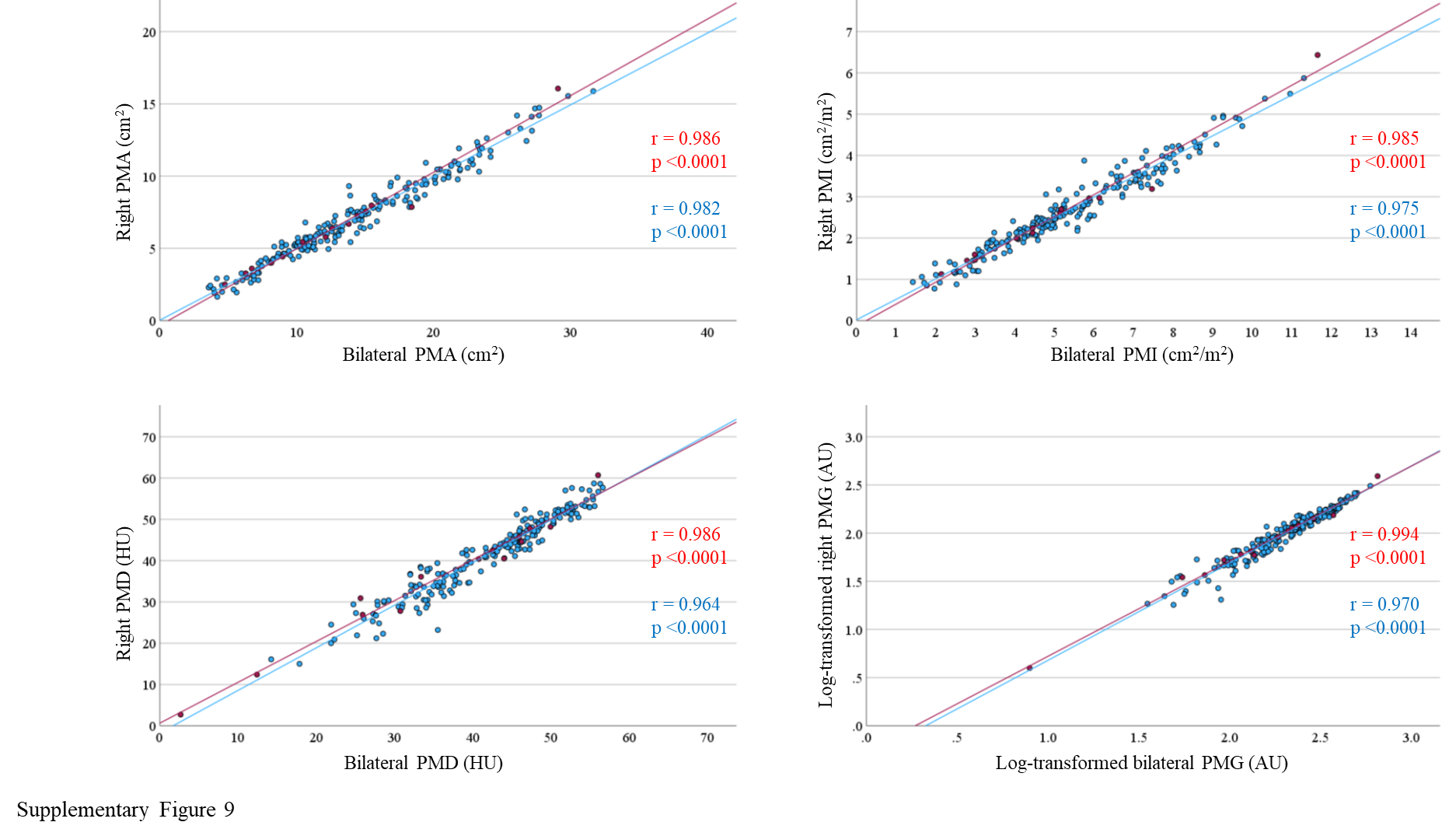

Supplement: Supplementary file 13 — Supplementary Figure 9. Correlations between the right and bilateral psoas muscle sarcopenic indices in patients with (red) or without (blue) lumbar spinal stenosis or hip osteoarthritis. AU, arbitrary unit; HU, Hounsfield units; PMA, psoas muscle area; PMD, psoas muscle density; PMG, psoas muscle gauge; PMI, psoas muscle index. (TIFF 400 kb) [file 40620_2025_2450_MOESM13_ESM.tiff]

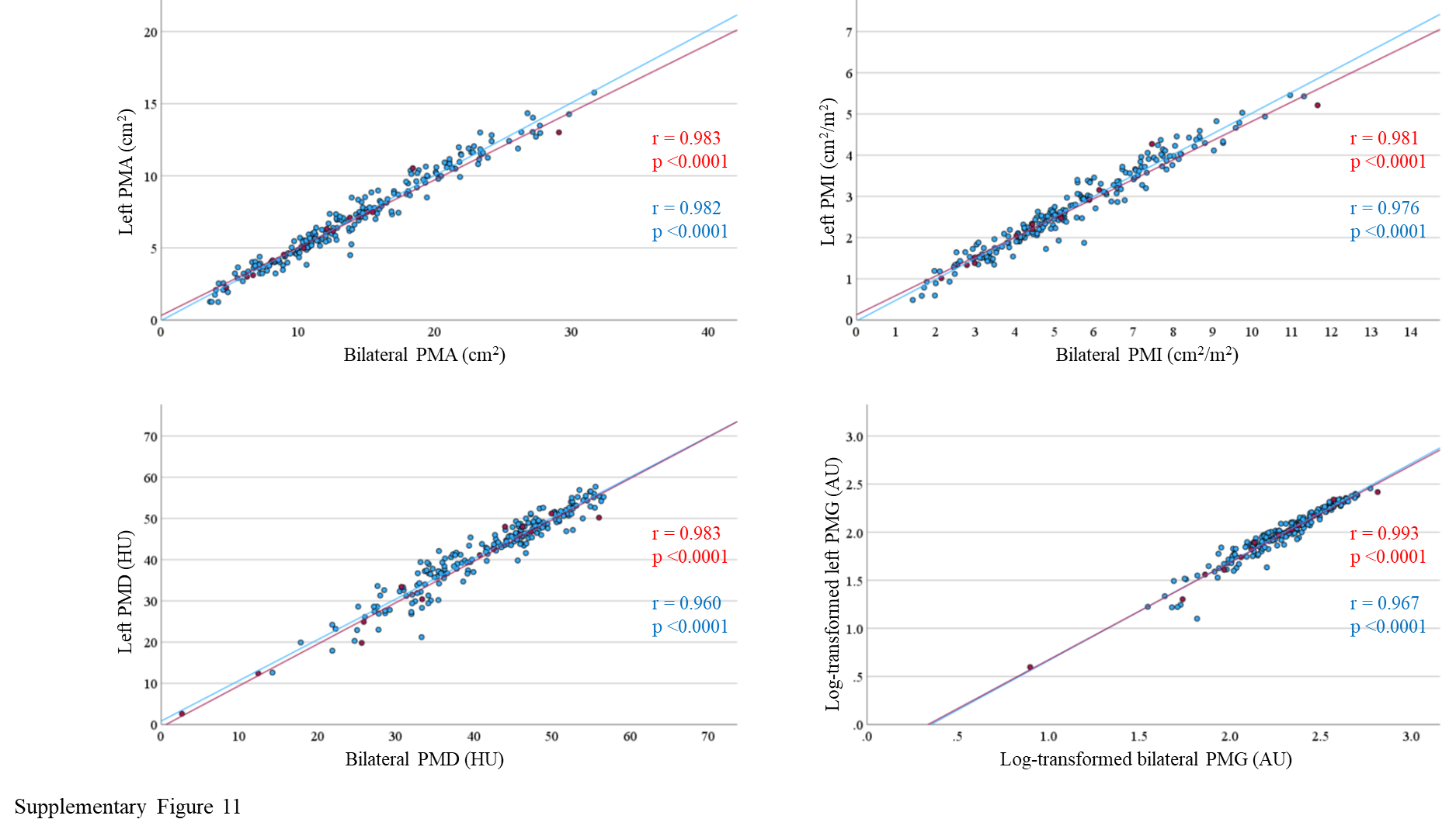

Supplement: Supplementary file 15 — Supplementary Figure 11. Correlations between the left and bilateral psoas muscle sarcopenic indices in patients with (red) or without (blue) lumbar spinal stenosis or hip osteoarthritis. AU, arbitrary unit; HU, Hounsfield units; PMA, psoas muscle area; PMD, psoas muscle density; PMG, psoas muscle gauge; PMI, psoas muscle index. (TIFF 394 kb) [file 40620_2025_2450_MOESM15_ESM.tiff]
